# A comparison study of sensory processing in older adults with and without dementia

**DOI:** 10.55730/1300-0144.5948

**Published:** 2025-02-02

**Authors:** Medine Nur ÖZATA DEĞERLİ, Didem KARADUMAN, Burcu Balam DOĞU, Onur ALTUNTAŞ

**Affiliations:** 1 Department of Occupational Therapy, Faculty of Health Science, Hacettepe University, Ankara, Turkiye; 2 Division of Geriatric Medicine, Department of Internal Medicine, Faculty of Medicine, Hacettepe University, Ankara, Turkiye

**Keywords:** Dementia, older adults, sensory processing

## Abstract

**Background/aim:**

Sensory processing is affected by aging, and dementia may lead to changes in sensory processing. This study aimed to examine the differences in sensory processing and sensory modulation between older adults with and without dementia.

**Materials and methods:**

This study was designed as a comparative study of community-dwelling older adults aged 65 years and older with and without dementia. Participants and their caregivers were recruited at the occupational therapy clinic of a public hospital. They completed a sociodemographic information form and the Adolescent/Adult Sensory Profile to assess sensory processing and sensory modulation.

**Results:**

The study included 120 older adults (60 with dementia and 60 without dementia) aged 75.35 ± 7.86 years. Older adults with dementia showed an above-norm low registration (*x*^2^ = 27.62) and sensory sensitive (*x*^2^ = 14.96) compared to without dementia. However, no significant differences were observed between the groups in sensation seeking and sensation avoiding (p > 0.05). There was a difference between the two groups in taste/smell, movement, visual, touch, and auditory modulation (p < 0.05).

**Conclusion:**

These findings highlight significant differences in sensory processing and modulation associated with dementia. Understanding these differences may offer valuable insights for developing individualized approaches to dementia management.

## 1. Introduction

Sensory processing is a neurological process that enables the encoding, interpretation, and recall of sensory stimuli from the body and environment to facilitate appropriate response [1–3]. Sensory modulation refers to the brain’s ability to regulate which sensory inputs, when, and to what extent they are processed [4]. Dunn’s Model of Sensory Processing is based on neurological threshold and self-regulation [5–6]. The neurological threshold is the point at which a sensory stimulus is perceived, with thresholds ranging from low to high [7]. Individuals with low thresholds quickly recognize stimuli, while those with high thresholds may miss standard sensory inputs [8–9]. Self-regulation involves regulating sensory stimulation and can be either passive or active. Passive self-regulators do not take action to regulate sensory input, while active self-regulators employ strategies to control the type or amount of stimulation [10]. Individuals may respond differently to daily sensory stimulation, leading to a wide range of variations [11–12]. Although differences in sensory processing are normal, high degrees of variation may be associated with different psychopathologies [13]. Therefore, examining sensory processing and modulation in clinical populations is essential [14, 15].

Aging, along with degenerative processes in the brain and sensory systems, can impair the ability to process and respond to sensory information, putting older adults at risk for sensory processing problems [16]. Older adults commonly experience visual (e.g., acuity, clarity, contrast sensitivity) and hearing problems (e.g., auditory localization, identification, discrimination) [17]. They also face reduced sensitivity to taste and smell, tactile discrimination difficulties, and balance issues, increasing the risk of falls [18, 19]. The vestibular sensory system, which adjusts head and body position by gravity, is also affected in old age; the most common examples are the inability to control balance and posture and frequent falls [20]. Decreases in proprioceptive sensory awareness, which refers to the position changes in muscles and joints that enable the perception of the body’s position in space, are also among the sensory risks faced by older adults; they may have difficulty establishing relationships between body parts [21]. These sensory changes limit the stimuli received from the environment, yet the processes of sensory modulation in older adults remain underexplored [22, 13].

Sensory processing difficulties, which are common in older adults, may be related to age and dementia [7, 23]. However, the sensory processing of older adults with dementia has been largely overlooked [24, 25]. Many studies suggest that sensory differences observed in individuals with dementia, such as low vision and hearing, could serve as biomarkers for early diagnosis [7, 26]. These studies, however, tend to focus only on specific sensory areas and do not examine the broader process of sensory processing. Sensory processing and modulation difficulties in older adults, particularly those with dementia, have not been adequately explored in the literature [27]. Therefore, it has been suggested that evidence of sensory differences in older adults with dementia be examined [23]. Studies comparing sensory processing and modulation in older adults with and without dementia are needed [28]. This study aimed to compare sensory processing skills and modulation in older adults with and without dementia.

## 2. Materials and methods

### 2.1. Study design

This study was designed as a cross-sectional comparison study. It was conducted in accordance with the Declaration of Helsinki, and ethical approval was obtained from the Hacettepe University Non-Interventional Ethics Committee (GO 21/971). The individuals who agreed to participate in the study provided written informed consent.

### 2.2. Participants

Sixty individuals with dementia were included in the study group, and sixty individuals without dementia were assigned to the control group. The sample size was determined using the G*Power 3.1.9.4 program, based on an effect size of 0.20 (Cohen’s small effect size), with 80% statistical power (β = 0.80) and a 5% margin of error (α = 0.05), accounting for potential dropouts. Consecutive patients presenting to the geriatric medicine outpatient clinic for comprehensive geriatric assessment were enrolled in the study.

The inclusion criteria for the dementia group were being 65 years of age or older, having a diagnosis of dementia according to the DSM-V, and experiencing moderate cognitive decline based on the Global Deterioration Scale (Stage 5) and the Mini-Mental State Examination (score of 10–19) [29–31]. Additionally, participants had not previously participated in any intervention targeting sensory processing, such as sensory integration therapy or sensory-based environmental modification. Information regarding the evaluation process for individuals with dementia was obtained not only from the patients themselves but also through the active involvement of their caregivers or cohabitants throughout the assessment. The inclusion criteria for the control group without dementia were being 65 years of age or older, having no any diagnosed cognitive impairment, and demonstrating healthy cognitive function according to the Mini-Mental State Examination (score of 24 or higher) [32]. For both groups, the exclusion criteria were visual defects that could not be corrected with spectacles, hearing loss, neurologic disease diagnosis, or orthopedic disabilities.

### 2.3. Instruments

#### 2.3.1.Socio-demographic Information Form

Participants’ age, sex, education level, and number of children were collected using a form developed by the researchers.

#### 2.3.2. Adolescent/Adult Sensory Profile (AASP)

The AASP is used to assess individuals’ sensory processing skills and modulations [33]. It consists of six sub-dimensions and 60 items that evaluate the level of taste/smell, movement, visual, touch, auditory, and activity level experienced daily. The 60 items of the profile are equally divided into four quadrants, each containing 15 items related to different sensory processing patterns: low registration, sensation seeking, sensory sensitivity, and sensation avoiding [34]. Using a five-point Likert scale, ranging from 1 (almost never) to 5 (almost always), participants are asked to rate how frequently they respond to the sensory event/experience described in each item. The score in each category ranges from 5 to 75 points. The higher the score, the more characteristic the individual’s sensory processing patterns are. Participants are then classified into four quadrants using the following expressions: “much more than most people”, “more than most people”, “similar to most people”, “less than most people”, and “much less than most people”. The phrase “similar to most people” in the survey refers to the general population that does not experience issues with sensory processing. The Turkish validity and reliability study of the scale was conducted by Üçgül et al., and it has a high internal consistency (r = 0.66–0.82) and test-retest reliability (0.67–0.82) [35].

Low registration is characterized by a combination of a high neurological threshold and passive self-regulation. Individuals with low registration process sensory input passively and do not actively seek additional sensory stimulation to meet their needs. These individuals often face challenges in understanding and expressing their internal emotional states and in interpreting the emotions of others. Sensation seeking, on the other hand, involves a high neurological threshold combined with active self-regulation. Such individuals enjoy an intense sensory environment and have an active behavioral strategy to provide this environment. Their motor behavior increases as they seek strong sensory inputs, such as loud sounds, vibrant visual stimuli, and intense tastes. Sensory sensitivity consists of a combination of low neurological and passive self-regulation. They are sensitive to sensory stimulation but are exposed to them without active behavior. There may be increased emotional and biological stress reactivity to sensory stimulation. Sensation-avoiding consists of a low neurological threshold and active self-regulation. They are hypersensitive to sensory stimuli and experience discomfort. They may limit exposure to the stimulus by reacting actively behaviorally.

### 2.4. Procedure

The researchers provided detailed explanations regarding the purpose and methodology of the study to all participants. Individuals with and without dementia who met the inclusion criteria and provided informed consent completed the sociodemographic information form and the Adult/Adolescent Sensory Profile (AASP) at the occupational therapy clinic of a public hospital. The caregivers of individuals with dementia participated in the assessment process. Each assessment session lasted approximately 25 to 30 min.

### 2.5. Analysis

Data were analyzed using the IBM SPSS Statistics 26.0 package program. Descriptive statistics were made using frequency (n) and percentage (%) values for categorical variables and minimum (min), maximum (max), mean (mean) and standard deviation (SD) values for numerical variables. Kolmogrov-Smirnov values, Skewness-Kurtosis values and histogram graphs were analyzed to determine whether the variables were suitable for normal distribution. For normally distributed variables, the difference between the groups was compared with the Independent T-test, and for non-normally distributed variables, the difference between the groups was compared with the Mann-Whitney U Test. The difference between categorical variables was evaluated by Chi-Square Test (*x*^2^). Statistical significance was accepted as p < 0.05.

## 3. Results

The study included 60 older adults with dementia (mean age: 73.47 ± 6.65 years) and 60 older adults without dementia (mean age: 75.35 ± 7.86 years). The control and dementia groups were similar in age, sex, marital status, and number of children. The demographic characteristics of the participants are presented in Table 1.

According to AASP scale scores, individuals with dementia scored statistically significantly higher in low registration and sensation avoiding than control group (p < 0.05). There was a difference between the two groups in terms of taste/smell, movement, visual, touch, and auditory (p < 0.05). The comparison of the sensory processing skills of the dementia and control groups is presented in Table 2.

According to Dunn’s Four Quadrant Model of Sensory Processing, 61.7% of the control group showed normative values in low registration, while 76.7% of individuals with dementia showed low registration above the norm (*x^2^* = 27.62). Sensation-seeking was below the norm in both groups and was similar in this respect. In sensory sensitivity, 60.0% of the control group were within the norm, whereas 65.0% of dementia were above the norm (*x^2^* = 14.96). Sensation avoiding, similar to sensory seeking, both groups were at the norm values. The comparison of sensory processing of dementia and the control group according to Dunn’s Four Quadrant Model is presented in Table 3.

## 4. Discussion

This study, which compared the sensory processing skills and modulation of older adults with and without dementia, found that there are differences in the sensory processing processes of older adults with dementia, which is a neurodegenerative disease that is seen in addition to the aging process. The older adults with and without dementia in this study were below the norm in sensation seeking, and their sensation avoiding was within the norm. However, older adults with dementia had higher levels of registration and sensory sensitivities. This study emphasizes that older adults with dementia show differences in taste/smell, movement, visual, touch, and auditory compared to their healthy age-matched controls. Awareness of differences in sensory processing in individuals with dementia may play an essential role in managing dementia.

Sensory processing is a fundamental concept in organizing individuals’ daily activities. It has been widely studied in children and childhood clinical populations in general, but there are limited studies in adult populations, especially in older adults [34]. Hebert stated that sensory processing in individuals over 60 years of age differs significantly from that of young adult [36]. The inhibitory deficit theory suggests that aging impacts the ability to inhibit non-activity-related sensory stimuli, with older adults experiencing difficulty in suppressing distracting or irrelevant stimuli [37]. Furthermore, in Cohen-Mansfield’s dementia-biological model suggests that neurological changes may disrupt the normal perception and processing of sensory information in individuals with dementia [38]. This study highlights that cognitive conditions, such as dementia, contribute to differences in sensory processing. Exploring the sensory processing differences between individuals with dementia and healthy older adults can provide valuable insights into dementia management strategies [39].

Older adults with dementia recorded fewer sensory stimuli (low registration is above the norm). This difference explains why older adults with dementia are less able to recognize sensory stimuli than their counterparts of the same age. While older adults without dementia can register a standard sensory stimulation immediately, older adults with dementia require a higher stimulus level to develop any response. Moreover, although they experience sensory stimuli, when they reencounter these sensory stimuli, they react as if they have never experienced them before. For example, not looking when called or not noticing when someone enters the room may be related to this. Ben-Avi et al. associate low registration with difficulty in acquiring information about the environment; older adults with dementia have problems receiving stimuli from the environment [40]. In addition, apathy, slowness, and lack of motivation in responding to sensory stimuli may be observed in individuals with dementia [14]. Furthermore, older adults with dementia had high sensory sensitivity. Older adults show a standard sensitivity to stimulation, i.e., high sensitivity if the stimulus is high and low sensitivity if the stimulation is intense. However, individuals with dementia are disturbed by even low sensory stimulation. Nevertheless, they respond with passive behavioral responses [33]. This may make them vulnerable to adapt or show flexibility in unexpected situations [15]. Low registration and sensory sensitivity may also be related. For example, individuals with dementia show a high startle response to the doorbell ringing, as if hearing it for the first time every time, because they do not register this sensory stimulus. When they experience it again, they always react to it with a high level of sensitivity instead of a normal response. These reactions do not precisely aim to solve the problem and may cause behavioral and psychological problems seen in dementia, such as anger, anxiety, and aggression [41]. In addition, the results of our study showed that 30% of older adults without dementia showed above-normal low registration and 33% sensory sensitivity. Prospective studies are recommended to examine the cognitive functions of individuals with abnormal values in low registration and sensory sensitivity.

In this study, both older adults with and without dementia showed decreased sensation seeking. Lawton et al. and Pohl et al. showed that older adults have less sensation-seeking than younger adults [42, 43]. This is due to the fact that older adults often struggle to inhibit distracting or irrelevant stimuli in their environment, and therefore, do not require additional sensory input [22]. Similar to healthy older adults, individuals with dementia do not need any additional sensory stimuli.

All older adults in the study exhibited sensation avoiding within the norm. More than half of the older adults without dementia showed sensory avoiding within the norm. In contrast, nearly half of the individuals with dementia showed sensory avoiding above the norm, and about half showed sensory avoiding above the norm. This proves that individuals with dementia may show different avoiding behaviors towards sensory stimuli. It is suggested to examine different factors that may cause sensation avoiding. In addition, this study found differences between individuals with and without dementia in the modulation of taste, smell, movement, visual, tactile, and auditory senses. Although studies on the sense of taste are limited, it is stated that the taste functions of individuals with dementia are significantly impaired compared to healthy older adults [42]. In addition, Suto et al. highlighted that eating behaviors in individuals with dementia are influenced by changes in their sense of taste [44]. These studies support the results of our study, which detected changes in taste modulation, a perceptual dimension, in individuals with dementia compared to healthy older adults. Individuals with dementia are less sensitive to tastes than healthy older adults. They do not seek sharp tastes (bitter, sour, minty, spicy). Symptoms seen in dementia, such as decreased or increased eating, and picky eating may be related to taste modulation. The ability to smell declines with aging and a marked reduction is observed in older adults, especially in individuals with cognitive impairment [45]. Moreover, sensory and smell impairments are associated with the transition between healthy, mild cognitive impairment, and dementia [46]. In addition to the literature, this study revealed differences in the modulation of the sense of smell in individuals with dementia compared to healthy older adults. Individuals with dementia do not recognize and respond to distinct odors.

Appropriate spatial orientation, posture, and movement accuracy decline with age, leading to an increased incidence of falls and injuries among older adults [47]. Studies on movement accuracy in individuals with dementia are limited. Our study emphasizes that there is a different movement modulation between healthy older adults and those with dementia. For example, individuals with dementia show behaviors such as constantly tripping, bumping, and needing to hold on. Yesanthrarao et al. found that individuals with dementia had twice the history of falls compared to their healthy counterparts, which supports the results of this study [48].

Previous studies have suggested a link between vision loss and cognitive impairment [49–51]. In a longitudinal study, Kershaw et al. demonstrated that individuals with vision problems are at a higher risk of developing dementia [52]. In this study, individuals with dementia have a different visual modulation than healthy older adults. While healthy older adults do not experience any discomfort in visually crowded environments with bright and colorful objects, individuals with dementia are disturbed by these environments and generally prefer a dim and visually quiet environment.

In Bruhn and Dammeyer’s study, it was reported that touch sensation worsened in individuals with dementia compared to their peers [53]. In this study, the modulation of tactile sensation was different with and without dementia. For example, individuals with dementia did not hesitate to be in contact with people or touch anything. The literature supports this behavior towards touch sensation in terms of using tactile stimuli for relaxation in individuals with dementia [54].

Auditory impairment is frequently associated with cognitive decline in older adults [55]. This has been linked to increased cognitive impairment and has been suggested to be a differential diagnostic criterion for dementia [56]. While it is well established that hearing loss can distinguish healthy older adults from those with dementia, research on auditory processing remains limited [57]. This study emphasizes the difference in auditory modulation between dementia and healthy older adults. For example, individuals with dementia have difficulty inhibiting unnecessary auditory stimuli in noisy environments and cannot focus on the required stimulus. They are often frightened by unexpected noises or have difficulty following when someone speaks quickly. In contrast, healthy older adults show more adaptive behavior to different sounds.

This study has several limitations. Although dementia associated with lower levels of education and female sex, it was essential to compare individuals with homogeneous demographics. Another limitation of this study was the lack of documentation regarding the medications used by the participants, which could potentially influence sensory processing. Furthermore, a major limitation was that participants’ behavioral and psychological symptoms related to dementia were not taken into account. In addition, unlike in any quartile, the AASP did not include any cut-off values for different modulation of sense, so the senses could not be compared according to the norm. A comparison of the seven other senses in the sensory integration theory will be helpful in dementia management. Also, types of dementia were not considered in this study. Comparison of sensory processing processes in different types of dementia in future studies will contribute to the literature.

This study reveals significant differences in sensory processing skills between older adults with dementia and those without dementia. Although both without and with dementia groups were within the norm for sensation seeking and avoiding, older adults with dementia had higher levels of low registration and sensory sensitivity compared to others. These differences, especially in processing taste/smell, movement, visual, touch, and auditory are significant for older adults with dementia. These findings emphasize that understanding sensory processing differences in dementia management processes can be critical in developing effective interventions more appropriate to individual needs. Therefore, individualized approaches focusing on the sensory processing skills of older adults with dementia may help them to improve their quality of life and better manage their life processes.

## Figures and Tables

**Table 1:** Demographics.

	Control (n = 60)		Dementia (n = 60)		
	Mean ± SD		Mean ± SD		p
**Year of age**	75.35 **±** 7.86		73.47 **±** 6.65		0.74
**Mini Mental State Examination Test**	28.46 ± 1.18		14.25 ± 2.83		0.01
	**n**	**%**	**n**	**%**	***x*** *^2^*
**Sex**					
Female	42	70.0	40	66.7	0.47
Male	18	30.0	20	33.3	
**Marital status**					
Single	7	11.7	15	25.0	0.52
Married	53	88.3	45	75.0	
**Education**					
Illiterate or primary school	36	60.0	39	65.0	<0.01
High school and more	24	40.0	21	35.0	
**Number of children**					
None	5	8.3	7	11.7	0.92
1–2	25	41.7	26	43.3	
3–4	16	26.7	14	23.3	
5+	14	23.3	13	21.7	

n: Frequency; SD: Standard deviation; p < 0.05

**Table 2:** Comparison of sensory processing skills of dementia and control groups.

	Control (n = 60)		Dementia (n = 60)		Mann-Whitney U Test
Quadrants	Mean ± SD	Range	Mean ± SD	Range	p
Low registration	33.05 ± 7.44	21–49	55.15 ± 13.85	25–76	<0.01
Sensation seeking	38.50 ± 7.51	24–61	37.80 ± 8.13	20–58	0.82
Sensory sensitivite	39.08 ± 8.92	17–63	47.61 ± 13.12	23–69	<0.01
Sensation avoiding	38.36 ± 8.26	24–58	40.73 ± 11.55	22–63	0.29
**Modulation**					
Taste/Smell	21.81 ± 4.38	14–31	20.60 ± 3.78	17–30	0.04
Movement	20.23 ± 4.22	14–33	23.30 ± 3.41	17–26	<0.01
Visual	26.96 ± 4.87	17–38	23.91 ± 3.57	20–29	<0.01
Touch	28.00 ± 6.96	14–44	30.83 ± 3.66	14–39	<0.01
Activity level	25.66 ± 4.58	13–35	25.26 ± 1.41	24–28	0.25
Auditory	26.50 ± 5.68	14–38	32.48 ± 4.11	27–37	<0.01

**Table 3:** Comparison of sensory processing skills of dementia and control groups according to Dunn’s Four Quadrant Model.

	Below norm		Norm		Above norm		
Quadrants	Control	Dementia	Control	Dementia	Control	Dementia	*x* *^2^*
Low registration	5 (8.3)	-	37 (61.7)	14 (23.3)	18 (30.0)	46 (76.7)	27.62*
Sensation seeking	43 (71.7)	36 (60.0)	16 (26.7)	24 (40.0)	1 (1.7)	-	3.22
Sensory sensitivity	4 (6.7)	-	36 (60.0)	21 (35.0)	20 (33.3)	39 (65.0)	14.06*
Sensation avoiding	5 (8.3)	8 (13.3)	33 (55.0)	26 (43.3)	22 (36.7)	26 (43.3)	1.85
